# The checkpointkinase 2 (CHK2) 1100delC germ line mutation is not associated with the development of squamous cell carcinoma of the head and neck (SCCHN)

**DOI:** 10.1186/1477-5751-9-10

**Published:** 2010-12-25

**Authors:** Kathrin Scheckenbach, Galatia Papadopoulou, Thomas K Hoffmann, Adam Chaker, Henning Bier, Jörg Schipper, Vera Balz, Martin Wagenmann

**Affiliations:** 1 Department of Otorhinolaryngology, Head and Neck Surgery, Heinrich-Heine-University Düsseldorf, Germany; 2 Department of Otorhinolaryngology, Head and Neck Surgery, Technical University of Munich, Germany

## Abstract

**Background:**

The checkpointkinase 2 (CHK2) is part of the highly conserved ATM-CHK2 signaling pathway, which is activated in response to DNA damage, in particular after double strand breaks which can be caused by carcinogens like smoking. After induction of downstream targets, e.g. the tumor suppressor p53, its activation leads to cell cycle arrest and apoptosis. Recently, the presence of CHK2 germ line mutations, primarily the 1100delC variant, has been reported to be involved in carcinogenesis. The CHK2 1100delC variant results in a truncated protein which is instable and inactive. Carriers of this variant have been shown to have an increased risk to develop breast cancer and probably also other tumors. Our purpose was to investigate the role of CHK2 germ line mutations in patients with squamous cell carcinoma of the head and neck (SCCHN).

**Materials and Methods:**

We investigated 91 patients suffering from SCCHN including all tumor sites (oropharynx, hypopharynx, larynx) for the presence of the germ line mutation 1100delC by direct sequence analysis. Patients were characterized by their tumor localization, tumor stage, age, the presence of additional malignant tumors and predisposing carcinogens (smoking, alcohol abuse).

**Results:**

None of the patients, independently of the tumor site, age, the abuse of predisposing carcinogens, or the presence of other kinds of tumors, carried the CHK2 1100delC variant.

**Conclusions:**

The germ line CHK2 1100delC variant does not seem to have a major impact on the development of SCCHN.

## Background

Head and neck cancer is the fifth most common cancer in the world [1]. The tumor suppressor p53 is strongly involved in the carcinogenesis of these tumors and inactivated either by mutations or human papilloma virus (HPV) infection in most of the cases [2]. Furthermore, squamous cell carcinomas of the head and neck (SCCHN) are associated with smoking and alcohol consumption as risk factors for their development [3]. These genotoxic substances lead to DNA damage; in particular DNA double strand breaks that are removed by different DNA repair mechanisms in healthy cells [4]. Two main checkpoint pathways are initiated in response to DNA damage and lead to either apoptosis or cell cycle arrest to allow chromatin repair: the ATR (ataxia telangiectasia and Rad3 related)-CHK1 (checkpoint kinase 1) -pathway and the ATM-CHK2-pathway. The checkpoint kinase 2 (CHK2, CHEK2) acts as a signal transducer within the highly conserved ataxia telangiectasia-mutated (ATM) protein kinase - CHK2-signaling pathway.

[5-10] Germ line mutations of p53 are normally the hallmark of patients with Li-Fraumeni syndrome, who typically develop tumors at an early age of life at different sites. In 1999, Bell et al. described CHK2 germ line mutations in patients suffering from Li-Fraumeni syndrome or Li-Fraumeni-like syndrome [11] without a germ line p53 mutation. One of the most important mutations was the 1100delC deletion. This variant leads to a frame shift and encodes a premature stop codon within the catalytic domain. The resulting truncated protein is inactive and unstable [12]. The frequency of the 1100delC variant differs within various populations [5]. It was found in 0.9% of the Northrhine-Westphalia population [13].

The CHK2 1100delC variant has been associated with breast cancer in multiple-case families and has been linked to an approximately 2-fold increased breast cancer risk. Thus, CHK2 is considered as a „low penetrance gene" for breast cancer [14-18]. CHK2 mutations including the 1100delC variant have also been associated to an elevated risk for prostate [19-22] and bladder cancer [23]. A correlation between CHK2 mutations and colorectal cancer [20,24,25], thyroid cancer and kidney cancer is discussed [20]. CHK2 variants have also been found in other tumors of the lung, larynx, pancreas, stomach as well as melanoma [26], osteosarcoma [27], Non-Hodgkin lymphoma [28], myelodysplastic syndrome or acute myeloid leukemia [29]. However, no definite relation to increased cancer susceptibility was shown [20].

SCCHN are generally carcinogen-induced tumors that show a high rate of p53 inactivation. Therefore, we investigated the presence of the CHK2 1100delC germ line mutation as a potential predisposition for the development of SCCHN with special attention to multi-tumor patients, and patients who are at low risk for SCCHN with regard to age or carcinogen abuse.

## Methods and Patients

### Patients

The study consists of 91 consecutive patients with histologically confirmed SCCHN, including all sites (57 oropharynx, 12 hypopharynx, 22 larynx) and stages (T_1-4_, N_0-3_, M_0/1_) of disease. After obtaining informed consent, blood samples were taken from each patient. Research was carried out in compliance with the Helsinki Declaration. This study was reviewed and approved by the ethics committee of the University of Düsseldorf.

### Sequencing of the CHK2 exon 10 including the 1100delC variant

DNA was isolated from peripheral blood lymphocytes (Genomic DNA purification kit, *Genera *Biosystems, Minneapolis, USA). Exon 10 of the CHK2 gene was amplified in a standard PCR reaction using Qiagen Mastermix (Qiagen, Hilden, Germany) and primers (5'-GCA AAA TTA AAT GTC CTA ACT TGC-3', 5'-TCT GCC CAG ACT TCA GGA AT-3'). PCR amplification was performed as a "touch down PCR". It comprised 35 cycles subdivided into 3 cycles of denaturing for 15 sec at 94°C, annealing for 15 sec at 68°C, and extension for 45 sec at 72°C followed by 3 cycles of denaturing for 15 sec at 94°C, annealing for 15 sec at 63°C, and extension for 45 sec at 72°C followed by 3 cycles of denaturing for 15 sec at 94°C, annealing for 15 sec at 58°C, and extension for 45 sec at 72°C. The PCR was preceded by 3 min at 94°C and followed by 7 min at 72°C. The amplificates were purified (Qiaquick, Qiagen), and mixed with ABI PRISM BigDye Terminator sequencing kit (Applied Biosystems, Weiterstadt, Germany) and primers (5'CCA GAT TAA TGG CAG GTG TG-3' for sense direction or 5'CCT ACC AGT CTG TGC AGC AA-3'for antisense direction). After the sequencing reaction (25 cycles of 15 sec at 96°C and 4 min at 60°C), the products were gel-purified (DyeEx 2.0 Spin Kit, Qiagen) and analyzed with an automated sequencer (ABI 310, Applied Biosystems). A sample representing the wild type sequence of CHK2 Exon 10 served as control. All samples underwent confirmation by repeated analysis.

## Results

The 91 investigated patients with histologically confirmed squamous cell carcinoma of the head and neck (SCCHN) comprised 15 women and 76 men. Their age ranged from 32 to 82 years with a mean of 56 years. A relatively high proportion (12 patients) were aged under 40 years at the time of diagnosis and can thus be considered as young for the development of a SCCHN. The majority (57) suffered from oropharyngeal carcinoma, 12 showed hypopharyngeal carcinoma, and 22 had laryngeal carcinoma (Table 1).

**Table 1:** The table shows the number of patients with different tumor subgroups and tumor stages.

Tumor Stage	Oropharynx n = 57	Hypopharynx n = 12	Larynx n = 22
T1	16	1	10
T2	21	8	6
T3	15	1	3
T4	5	2	3

In 49 patients, cervical lymph node metastases were found while 42 patients showed no metastases. Distant metastases were determined in 3 patients. 21 patients also suffered from other malignant or semi-malignant tumors (Table 2). Some patients had tumors at multiple sites. One patient had a history of an esophagus carcinoma, a basal cell carcinoma, and a melanoma; another one suffered from a prostate and a bladder carcinoma, and one patient experienced a colon carcinoma and a basal cell carcinoma.

**Table 2:** The table shows number of patients suffering from second malignancy.

Secondary malignancy	Number of Patients suffering from a secondary malignancy
Esophagus	6
Lung	3
Basal Cell Carcinoma	4
Bladder	3
Prostate	3
Colon	2
Chronic myeloid leukemia (CML)	1
Chronic lymphocytic leukemia (CLL)	1
Melanoma	1

16 of the patients were non-smokers with a high proportion of 75% (12 out of 16) of patients showing an oropharyngeal cancer.

14 of those also were no habitual drinkers. Altogether, 23 patients reported moderate to seldom alcohol consumption (less than once a week). The remaining patients were mostly heavy smokers and drinkers.

Sequence analysis of exon 10 of the CHK2 gene was performed for all patients. However, none of the patients showed the presence of the 1100delC variant.

## Discussion

None of the investigated 91 patients with SCCHN carried the CHK2 1100delC variant. The incidence of 1100delC in the population of the state Northrhine-Westphalia in Germany is reported to be 0.9%. Because our investigation took place exactly in this region, we accepted the published data as a control group for our patients [13]. Hence, we did not determine any significant difference in the incidence of the CHK2 1100delC variant between the tumor group (0%) and the control group (0.9%). Compared to the study sizes of some other investigators, we only investigated a relatively small group of 91 patients. But if the CHK2 1100delC variant had a major impact for the development of SCCHN, at least some of the patients should have been positive for this mutation.

The heterozygous germ line mutation 1100delC of CHK2 was previously reported to be associated to breast cancer [18], bladder cancer [23] and prostate cancer [19-22] and perhaps also to other carcinomas [20,24,25]. Cybulski et al. analyzed multiple kinds of carcinomas for CHK2 germ line mutations. This study also included 245 laryngeal carcinomas [20]. In this group of patients, they did not detect any truncating mutation. Therefore, we were able to confirm these results. Nevertheless, they found the missense I157T mutation in 4.1% of the cases. In this study, the incidence of this variant within the tumor group did not significantly differ from the control group. Furthermore, Cybulski et al. more recently performed an additional investigation where they analyzed 895 cases of lung cancer, 430 cases of laryngeal cancer and 6391 controls for the I157T variant. They reported that the I157T variant appears to be associated with a decreased risk for developing lung cancer and laryngeal cancer [30]. In this case, CHK2 alterations may be not predisposing but protective for head and neck cancer. In our study, we did not screen our patients for this variant yet.

Until now, no data for two additional major tumor sites in the head and neck area, hypopharynx and oropharynx, were available for the risk of predisposing CHK2 mutations. In the present study, we did not find the CHK2 1100delC variant in any of these patients.

Moreover, patients suffering from multiple tumor types including squamous cell carcinoma of the head and neck, showed no CHK2 1100delC variant. This indicates that this particular germ line variation plays no significant role for the development of cancer of the upper aerodigestive tract.

However, CHK2 may play a role either in the defense or the carcinogenesis of these tumors. The ATM-driven DNA-damage pathway seems to be activated in due to tobacco smoke, a major carcinogen for the development of SCCHN, as Tanaka et al recently showed [31]. Because CHK2 is a major target of ATM, a smoking-dependent-CHK2 activation in SCCHN is likely. Yoon et al. investigated the expression of phosphorylated CHK2 (pCHK2) and therefore activated CHK2 in precancerous lesions of the oral mucosa immunohistochemically. He found that subjects with a positive pCHK2 staining had a significantly (8.6 fold) higher risk to develop a squamous cell carcinoma out of this lesion. He suggested pCHK2 as a putative biomarker for oral precancerous lesions [32]. However, the authors did not investigate the occurrence of CHK2 mutations. Serbia et al. investigated the pCHK2 status of squamous cell carcinomas of the esophagus in patients who underwent neoadjuvant chemotherapy (RTX) immunohistochemically. They described that pCHK2 positive tumors more frequently showed clinical regression after RTX [33]. Because esophageal cancer is closely related to the upper aerodigestive tract, a similar behavior might be assumed for SCCHN.

## Conclusion

The typical 1100delC germ line mutation does not seem to have a major impact on the risk to develop squamous cell carcinoma of the head and neck. Since this study is limited by a relatively low case number, additional studies including larger groups of patients should be performed. Furthermore, the detection of CHK2 variations other than 1100delC in SCCHN as well as the definition of the role of CHK2 in the carcinogenesis of SCCHN remain to be an interesting matter for future investigations.

## Abbreviations

ATM: ataxia telangiectasia-mutated protein kinase; ATR: ataxia telangiectasia and Rad3 related; BRCA1: breast cancer 1; CDC25A: cell division cycle 25 homolog A; CDC25C: cell division cycle 25 homolog C; CHK1: checkpoint kinase 1; CHK2: checkpoint kinase 2; E2F1: E2F transcription factor 1; FHA: forehead-associated domain; HPV: human papilloma virus; PIKK: phosphatidylinositol-3 kinase (PI-3K)-like kinase; PML: promyelocytic leukemia protein; SCCHN: squamous cell carcinomas of the head and neck

## Competing interests

The authors declare that they have no competing interests.

## Authors' contributions

KS performed most of the sequencing, isolated DNA, organized the study and wrote most parts of the article. GP collected the patient's samples and isolated DNA. MW wrote parts of the article, collected samples and investigated patients. AC and TKH collected samples and investigated patients. HB and JS corrected the article and investigated patients. VB wrote parts of the article, designed the primers and performed sequencing.

All authors read and approved the final manuscript.
